# P68 RNA helicase as a molecular target for cancer therapy

**DOI:** 10.1186/s13046-014-0064-y

**Published:** 2014-08-24

**Authors:** Ting-Yu Dai, Liu Cao, Zi-Chen Yang, Ya-Shu Li, Li Tan, Xin-Ze Ran, Chun-Meng Shi

**Affiliations:** 1 Institute of Combined Injury, State Key Laboratory of Trauma, Burns and Combined Injury, Chongqing Engineering Research Center for Nanomedicine, Department of Preventive Medicine, Third Military Medical University, Chongqing 400038, China

**Keywords:** RNA helicase, p68, Molecular target, Transcriptional co-activator

## Abstract

The DEAD-box family of RNA helicase is known to be required in virtually all cellular processes involving RNA, and p68 is a prototypic one of the family. Reports have indicated that in addition to ATPase and RNA helicase ability, p68 can also function as a co-activator for transcription factors such as estrogen receptor alpha, tumor suppressor p53 and beta-catenin. More than that, post-translational modification of p68 including phosphorylation, acetylation, sumoylation, and ubiquitylation can regulate the coactivation effect. Furthermore, aberrant expression of p68 in cancers highlights that p68 plays an important role for tumorgenesis and development. In this review, we briefly introduce the function and modulation of p68 in cancer cells, and put forward envisagement about future study about p68.

## Introduction

The DEAD-box family of RNA helicases contains nine domains of strong peptide sequence conservation, including the Asp-Glu-Ala-Asp (DEAD) helicase signature sequence. In addition to regulating the conformation of RNA structure and the known ATP-dependent RNA helicase activity [1], DEAD box-containing proteins are required for a variety of processes involving RNA like ribosome biogenesis, embryogenesis, spermatogenesis, and cell development and division [2].

P68 (DDX5) is considered as a prototypic member of the DEAD-box family of RNA helicases. It was fortuitously discovered through a cross-reaction with an antibody against the simian virus SV-40 T antigen, and shows extensive amino-acid sequence homology to eukaryotic translation initiation factor eIF-4A, the first identified helicase capable of unwinding RNA [3]. In no time, p68 was reported to be an RNA-dependent ATPase and has RNA-unwinding activity [4].

Many research studies demonstrated that p68 is important for a diverse range of cellular processes, including pre-mRNA, rRNA and miRNA processing and transcription [5]. Furthermore, the existence of p68 and the highly related DEAD box family member p72 can be found in a diversity of complexes in the cell, together with other factors [6]. There is a considerable body of evidence indicating that p68 is an important co-activator of transcription factors, for example, estrogen receptor ? (ER?) [7], MyoD [8], Runx2 [9], androgen receptor (AR) [10], and p53 [11], which have shown clear significance in cancer. Recent studies have also demonstrated that p68 is aberrantly expressed/modified in several types of cancers, suggesting that p68 plays important roles in cancer development and progression [12].

For an illuminating purpose for cancer therapeutic utilization, we summarize the structural and functional characteristics of p68 in both biological and pathological conditions, which will highlight the potential target for anticancer therapy.

### Structure characteristics and biological functions of p68(DDX5)

**Figure 1:**
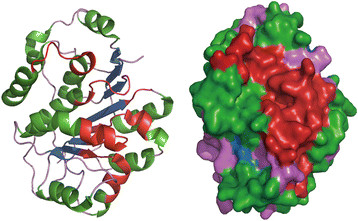
**Stereoview of secondary structure of the N-terminal domain of the human DEAD-box RNA helicase DDX5(P68). left**: ribbon and helix diagram; **right**: surface representation. Different secondary structures are marked by different colors. The conserved motifs are colored red. They are successive and converge on one side of the surface of the overall structure. The functional area can be modified or combined with other proteins. (PDB ID: 4A4D).

P68 shares a ¿helicase core¿ of nine conserved motifs with other members of the DEAD-box family, and these conserved regions are critical for RNA binding, ATP binding and hydrolysis, and intermolecular interactions [13]. The core is divided into two flexibly linked RecA-like domains, Domain 1(D1) and domain 2(D2). The D1, consisting of Q-motif, motifs I, II and III, serves for ATP-binding. The D2, including motifs IV, V and VI, exhibits an RNA-duplex recognition domain [14]. The Q-motif is present at the N-terminus of the catalytic core and is preceded by a conserved phenylalanine 17 amino acids at the upstream [15]. This conserved aromatic group and the Q-motif are identified as adenine recognition motif and can regulate ATP binding and hydrolysis [16]. Further, the Q-motif was reported to affect the helicase activity through regulating the affinity between the protein with RNA substrates [17]. Motifs I and II (or Walker A and B) are capable of binding ATP. The energy from ATP hydrolysis is coupled to RNA unwinding by motif III and cooperates with other motifs to create a high-affinity RNA binding site [18]. And motif IV, together with motif Ia, Ib and V, is engaged in ATP-dependent binding of RNA substrates [19]. Obviously, the structure of p68 is in a dominant position in the representation of function, and there is a profound work which is remained to elucidate the mechanism between structure and biological activity [20] (Figure 1).

As is known to all, p68 is a prototypic multifunctional protein, and the most well recognized function of p68 is to bind both double- and single-stranded RNA and provide energy to perform bidirectional RNA duplex unwinding activity as an ATPase [21]. Another important function of p68 is for efficient spliceosome assembling and RNA splicing [22]. Previous investigations demonstrated that p68 could unwind and separate the connection between the U1 small nuclear ribonucleoprotein particle and the 5¿ splice site, facilitating the dynamic formation from pre-spliceosome to spliceosome [23]. However, the assembly of the spliceosome is in an RNA helicase-independent manner [24]. RNA helicase p68 could also manifest conformational change activity enhancing the U1-5¿ss interaction [25]. Based on the observation, the p68 is important for RNA maturation. In succession, alternative splicing can also be regulated in this way [26].

MicroRNAs(miRNAs) are small non-coding RNAs that can affect cell development through regulating protein expression or messenger RNA synthesis [27]. The primary miRNA (pri-miRNA) is cleaved by the nuclear RNase III endonuclease Drosha in the cell nucleus and transitions to precusor miRNA (pre-miRNA) which in sequence will be transported to cytoplasm [28]. Afterwards, the pre-miRNA is processed by another nuclear RNase III endonuclease Dicer into mature double-strand miRNA. The Drosha forms two multi-protein complexes, and experimental results revealed that p68 is a component of the larger one [29]. Moreover, p68 can function as a recognizer of pri-miRNA and bind to the specific structure [30]. It suggests that p68 is required in the early stages of miRNA biogenesis involving the processing of pri-miRNA to pre-miRNA. Recently, much work has been done in the field of miRNA processing regulated by Drosha. The transforming growth factor ?(TGF?) signal transducers, Smad protein, as well as the tumor suppressor p53, has been reported to recruite into Drosha by interaction with p68, facilitating accumulation of Drosha complex to specific pri-miRNA [31]. However, the Smad and p53 are probably in competition for p68 binding and thus maturation of miRNA is dynamically modulated by these proteins [32].

It also has been indicated that p68 is required in rRNA processing [33]. In the early stage of ribosome biogenesis in nucleolus, p68 is related to the restructuring of 32S pre-rRNA [34]. And in the following steps, the p68 is exported from nucleoli and has been shown to be associated with nucleoplasmic processing of 5.8S pre-ribosomes [30]. Nucleophosmin (NPM) (B23) is a nucleolus/nucleus-cytoplasm shuttling protein and is indispensable in cell development and proliferation. The fundamental function of NPM is to transport ribosomes and ribosomal subunits from nucleus to cytoplasm, and p68 is an important binding protein of it [35]. The tumor suppressor ARF, which inhibits cell cycle, has been considered as a hinder of exportation to suppress rRNA biogenesis by associating with NPM [36]. Consistent with the appearance, the ARF is also a blocker in the interaction between p68 and NPM, and effects the production of mature rRNA [37].

### Abnormal expression of p68 in cancer

Since p68 is ubiquitously expressed in human tissues and plays a multifunctional role in a number of the cellular processes, many experimental results revealed examples of diseases concerned with p68, including obesity [38], Down syndrome [39], myotonic dystrophies [40] and especially cancer [5]. Over the past few decades, abnormal expression of p68 has been detected in many cancers, such as colon cancer [41], breast cancer [42], lung cancer [43], cutaneous squamous cell carcinoma [44], leukemia [45] and so on (Table 1).

**Table 1:** Aberrant expression of p68 in cancer

**Type of tumor**	**Expression level of p68**	**Involved factor**	**Author/reference**
**Colorectal cancer**	elevated in HCT-116, LoVo, SW480 and SW620 cell lines relative to normal colon cell lines	?-catenin, p21	Singh C *et al.,* 1995 [41]
			Shin S *et al.,* 2007 [46]
	higher p68 positive rate of adenocarcinoma than matched normal tissue, and the expression degree increases from polyp to adenoma and adenocarcinoma in sequence		Causevic M *et al.,* 2001 [47]
**Breast cancer**	increased progressively from the luminal to basal breast cancer cell lines(38 cell lines)	miR-182,ER?	Haines GK *et al.,* 1996 [42]
	elevated in cancer tissue than in normal breast tissue		Wang D *et al.,* 2012 [48]
	higher DDX5 positive rate in malignant biopsies than benign		Fujita T *et al.,* 2003 [49]
**Head and neck squamous cell carcinoma**	elevated in (UMSCC)-10B and (UTSCC)-19A cell lines compared two benign epithelial keratinocytes	Not determined	Beier UH *et al.,* 2006 [50]
**Prostate cancer**	higher p68 positive rate in PCa biopsies than in BPH	AR	Clark EL *et al.,* 2008 [10]
**Salivary gland pleomorphic adenomas**	elevated in adenoma than matched normal salivary gland tissue	Not determined	Zhang X *et al.,* 2009 [51]
**Leukemia**	higher level in SupT1, DND41, HBP-ALL, KOPT1, Jurkat, MOLT4, MOLT13, MOLT15 and REX cell lines than selected acute myeloid leukemia cell lines	NOTCH1, MAML1	Lin S *et al.,* 2012 [45]
	elevated in human T-ALL bone marrow samples compared with normal T cells		
**Glioma**	elevated in H-4, HS-683, U-87, U-251, and U-343 cell lines	NF-kappaB, p50	Wang R *et al.,* 2012 [52]
	elevated in high-grade human glioma relative to low-grade glioma and normal adjacent brain tissue		
**Cutaneous squamous cell carcinoma**	elevated in carcinoma than adjacent tissues and normal foreskin tissues	Not determined	Wang SJ *et al.,* 2012 [44]
	higher in carcinoma cases with metastasis than those without metastasis		
**Hepatocellular carcinoma**	down-regulated in HBV-positive and NBNC HCC tissues compared with matched adjacent non-cancerous liver	Not determined	Kitagawa N *et al.,* 2013 [53]

A clinical cohort study on prostate cancer (PCa) showed that the p68 expression in the PCa biopsies was markedly higher than matched benign prostatic hyperplasia (BPH) [10]. It is consistent with previous work, which also provided analogous comparison result between colon cancer and normal tissues, and this study further showed that defect in proteasomal degradation contributes to p68 accumulation [47]. And then, an additional research showed that post-translational modification, like sumoylation and ubiquitylation, would strengthen the stability of p68 [54]. Dysregulation of p68 expression may influence the miRNA processing machinery and promote benign tumor development [51]. And in cancers, the up-regulation of p68 is detected in both invasive and periphery normal tissues, suggested its early occurrence during tumor development [47]. Moreover, some experimental results indicated that the expression is associated with poor prognosis and probably resistance to therapy [52],[53], which highlight the possible role of p68 in selection of anticancer therapy and prediction of overall survival.

### P68 as a transcriptional co-activator in tumor development

P68 is also an important transcriptional regulator, acting both as a transcriptional co-activator for a diverse range of transcription factors including estrogen receptor ?(ER?) [55], the tumour suppressor p53 [11] and the myogenic regulatory factor MyoD [56]. Meanwhile, in other conditions, as a promoter-dependent transcriptional repressor [57].

### Estrogen receptor co-activator

The estrogen receptor ? is a member of the nuclear hormone receptor family of transcription factors that is activated by estrogen, and can regulate mammary gland development. Much work has been reported recently that ER? contributes a lot to the development and progression of breast cancer [58]. SRC-1/TIF2 family proteins is a part of the nuclear receptor AF-2 co-activator complexes with CBP/p300 and an RNA co-activator, SRA. And it is reported that p68/p72 can directly bind the SRC-1/TIF2 family proteins and interact with p72/p68 [59]. In addition, another study showed that p68/p72 can cooperate with SRC-1 and interact with ER? in an estrogen-independent manner [60]. Based on these reports, p68/p72, in synergy with ER?, CBP/p300, MyoD, can form a component of the ER? transcriptional complex. This indicates that p68 and p72 are important components of the transcription machinery. So further study of p68 will provide more evidences of p68 playing an important role in breast cancer development and/or progression.

### Androgen receptor

The androgen receptor (AR) belongs to the nuclear steroid hormone receptor family. It is an androgen-dependent transcription factor which play an essential role in the development and progression of prostate cancer (PCa). A number of androgen receptor (AR) transcriptional co-regulators have been identified to play important roles in prostate cancer (PCa). The p68 possess a LxxLL motif which is observed in cofactors that interact with ligand-activated nuclear hormone receptors [61]. What¿s more, p68 was found to be over-expressed in prostate cancers, to interact with AR, enhance AR transcriptional activity in luciferase reporter assays and to be recruited to the AR-responsive prostate specific antigen (PSA) promoter in the presence of RNAP II [62]. P68 siRNA knockdown also resulted in decreased expression of AR-responsive genes [10]. The observation demonstrate that p68 is a co-activator of the AR, independent of the p68 helicase function and c-Abl activity. So the abnormal regulation of p68 may affect the function of AR influencing the development and progression of prostate cancer.

### P53

P53 is now well established as an anti-oncogene, and p68 was found to be recruited to p53-responsive promoters in response to DNA damage, and also to the p21 promoter facilitating transcriptional initiation [11]. This would up-regulate the level of p21. Meanwhile, the acceleration of p21 results in more forming of cyclin D1/Cdk2 complexes, which can phosphorylate the retinoblastoma protein (pRb) [63]. Rb works as a regulator of the cell cycle, controlling passage through G1 phase [64]. And the unphosphorylated Rb would combine with the E2F transcription factors to block cell cycle [65]. It is consistent with previous work, which showed that p68 is over-expressed in cancer cells. Taken together, it seems that the p68 could regulate the cancer cell cycle.

P53 has multiple splicing variants. ?133p53 is one of the protein isoforms from mRNA variants, while it turns to negatively regulate the apoptosis mediated by p53 [66]. Experiments in breast cancer indicated that the expression of ?133p53 is up-regulated in p53-independent manner with p68 siRNA knockdown, and could inhibit the ability of p68 to coactivate p53-dependent induction to the cell cycle inhibitor p21 [67]. Recently, an additional research showed that p68 plays an essential role in recruitment of p53 to the p21 promoter, and selection of p53 function [68]. Taken together, we can construct a dynamic interaction among p68, p53 and p21. As we mentioned before, p53 could recruit to the Drosha complex through the association with p68 and facilitates the processing of pri-miRNAs to precursor miRNAs. And another study further shows that the recruitment forms through a carboxy-terminal half of the central DNA-binding domain [31]. Based on previous work, we can suppose that p68 may contribute to the carcinogenesis by interact with p53, Drosha and other factors in the procedure.

### ?-catenin

?-catenin is a protein that can modulate both cell adhesion and gene transcription, and p68 may regulate the ability of ?-catenin in different ways. It has been reported that p68 can form complex directly with ?-catenin through its helicase domain and facilitate the transcription activation of ?-catenin [46]. Besides, the phosphorylated p68 could regulate the transcription of ?-catenin downstream effectors including c-Myc, cyclin D1 [69]. It also has been shown that the Wnt binds to receptors and co-receptors initiating the translocation of destruction complex, and nuclear localization of ?-catenin [70]. And p68, cooperated with mrhl RNA, negatively regulated the Wnt signaling [71]. So it suggested that p68 plays an important role in the stabilization of ?-catenin level. Recently, several work has been reported in the field of the p68-?-catenin signaling. For example, the platelet derived growth factor (PDGF), which is important for the development of prostate cancer, was reported to activate p68-?-catenin signaling in PCa cells [72]. Experiments also indicated that the p68 is positively correlated with endogenous ?-catenin and modulate PDGF-BB induction of Mcl-1 (myeloid cell leukemia-1) expression [73].

Smad protein is an important signal transducer of TGF-beta, and the TGF-beta signaling can promote some specific miRNA maturation through the binding of Smad protein to the Drosha complex component p68 [32],[74]. Recently, it has been discovered that the mesenchymal to epithelial transition(MET) can be induced in response to TGF-beta [75]. The mechanism is based on the interaction of some mesenchymal activators, including ?-catenin, and the Smad protein to form an EMT promoting Smad complexes (EPSC) in tumors [76]. Consistent with the outcome, there were some other works demonstrated that the ?-catenin-Smad complex could signal as a regulator to TGF-beta induced EMT [77],[78].

### Post-translational modification of p68 in cancer development and progression

Experimental data have indicated that the protein kinase C(PKC) can phosphorylate p68 and the latter binds to calmodulin in a Ca2?+?? dependent manner [79]. Now it is clearly that the p68 RNA helicase is a member of the IQ domain-containing family and the C-terminal domain of p68 is a substrate for PKC in the absence of RNA. But both phosphorylation and calmodulin binding are suppressors for p68 ATPase activity. And documents further showed that the C-terminal domain of p68 RNA helicase binds ssRNA [80]. In addition to the conserved motifs, there was an RGS¿RGG motif identified as an RNA¿binding domain of p68 [81]. Moreover, different phosphorylated residues may cause different decision on effects [82]. In conclusion, the main emphasis is placed on the topic on high correlation between phosphorylation of p68 and cancer development and progression, and more research is still required for the future diagnosis and therapy for cancer.

Post-translational modifications can extremely increase the diversity of protein in the progress of protein biosynthesis. Ubiquitylation, sumoylation and acetylation are also dominating modes for p68 modifications. Go on with the previous work, the over-accumulated p68 in variant cancers is then detected to be poly-ubiquitylated, and the expression distinguish between normal and tumor cells indicate that the ubiquitylation of p68 can be important for the tumorgenesis [47]. Meanwhile, sumoylation of p68/p72 is also upregulated. The sumoylation is considered as a multiple-effect modification. It is reported that SUMO (small ubiquitin-related modifier) modification can increase the stability of p68 and promote the binding to the acetyltransferase p300, furthermore, to regulate the activity of p53 and estrogen receptor [83]. Besides, SUMO modification is on a relative fixed site (K53) of p68, and can in bi-direction regulate its transcriptional repression activity and the ability as a co-activator of p53 [84]. In addition, both p68 and p72 can bind to the acetyltransferase p300, and the acetylation mediated by p300 resulted in aberrant activation of p68, promoting the interaction with HDACs [83]. We can conclude that the regulation and modification of p68 may be a way to mediate the tumor development.

### Emerging roles of p68 in cancer development and progression

p68 is a promising research target for cancer, and the topic is investigated quite intensively in recent years. In addition to distinction in the expression of p68 between cancer and matched normal tissues, the expression level also varies with cancer cells in different states or degrees. For example, in cutaneous squamous cell carcinoma, the cancer tissue with metastasis has significantly higher than cases without metastasis [44]. Another documentation is the progressive increase of p68 expression level during the transition from polyp to adenoma, and then to adenocarcinoma in colon [46]. These details suggested that the RNA helicase p68 may play a vital role in regulating cancer cell grade and invasive potential.

**Figure 2:**
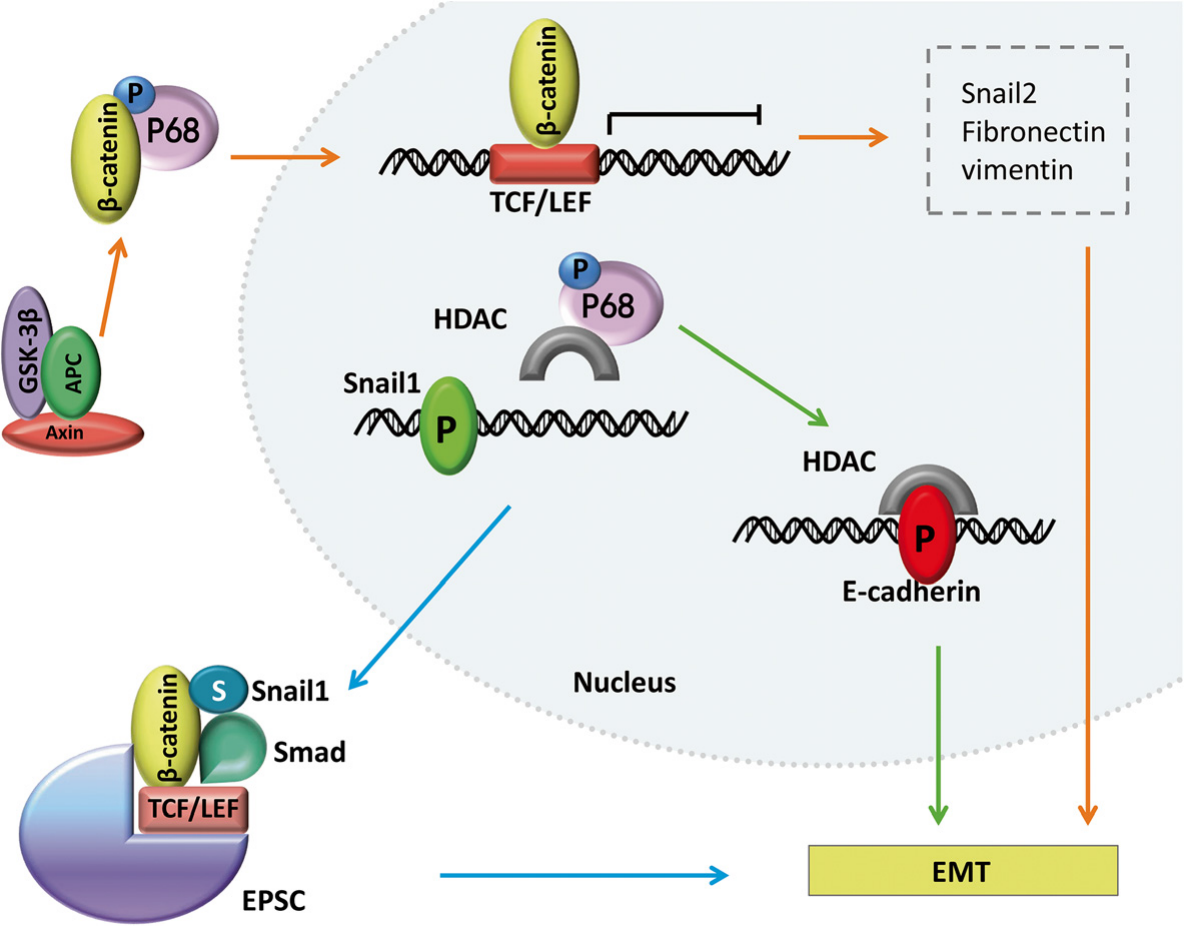
**The mesenchymal to epithelial transition(MET) and p68.** (orange line) The phosphorylated p68 dissociated ?-catenin from Axin destruction complex to nucleus, blocking the transcription of TCF gene, and regulating EMT related genes such as Snail2, fibronectin and vimentin. (green line) The phosphorylated p68 deconstructed the interaction between HDAC and Snail1 promoter, facilitating the binding of HDAC to the promoter of E-cadherin and blocking its transcription. (blue line) The destruction promoted the transcription of Snail1 gene. The Smad protein, associated with TCF, Snail1, ?-catenin and some other transcriptional factors to form EPSC in tumors.

The phosphorylation of p68 on Y593 contributes to the dissociation of ?-catenin from the Axin destruction complex and translocation to nucleus, promoting EMT, which is always considered as an initiation of invasion and metastasis [85],[86]. Further, the remodeling of cytoskeleton to form lamellipodia and filopodia is an essential step in migration progress. Experiment demonstrated that p68 could promote the deformation by cooperating with calmodulin, increasing the motility of cells [87]. Another previous experimental data also indicated that DDX5 plays a role in reorganization of actin cytoskeleton in breast cancer [48]. Then we can figure out that p68 is involved in preparing fundamental conformational change for migration cells. p68 could also regulate invasiveness of cancer cells by interacting with other factors, including COUP-TFI in breast cancer [88] and a specific RNA aptamer in colon cancer [89]. A class of anticancer drugs histone deacetylase inhibitors (HDACIs), was found to have the ability to induce tumor cell EMT via the over-accumulation of Snail [90]. Recent study showed that phosphorylation of p68 at Y593 facilitate the deconstruction between histone deacetylase (HDAC)1 and Snail1 promoter, initiating the transcription of Snail1 gene [91]. Then the upregulated Snail1 could block the transcription of E-cadherin by recruiting HDAC to the transcriptional promoter [92]. Combined with the observation on ?-catenin, we can figure that phosphorylation of p68 regulate cancer development and metastasis potential (Figure 2).

Drug resistance is another important feature of cancer cells [93]. It also has been reported that RNA helicase p68 is phosphorylated at tyrosine residue in cancer cells, compared with matched normal tissue, and the treatment of TNF-alpha and TRAIL (Tumor necrosis factor-related apoptosis-inducing ligand, an anticancer agent) would weaken the effect of phosphorylation [94]. In succession, the team demonstrated that the phosphorylation of p68 on some specific residues mediates the effect of apoptosis agents [95]. For example, a double tyrosine phosphorylation of p68 at Y593 and Y595 induced by PDGF can extenuate resistance to apoptosis induced by TRAIL [96]. Therefore the phosphorylated p68 may have a certain protective effect on the activities of cancer cells.

With so many emerging implications of RNA helicase p68 in tumorgenesis and progression, we can demonstrate that p68 could be a potential target for cancer therapy.

## Conclusion

As we have known, the modulation between p68 and the transcription factors may be involved with cancer metastasis potential and anticancer drug resistance. Unfortunately, although a lot of effort has been spent on the problem, the mechanism is still not so clear. In conclusion, we emphasize the possible oncogenic function of p68. Moreover, the niche-targeting regulation of p68 activation and expression may contribute to the possibility of blocking tumorgenesis and reinforcing the sensitivity of cancer cells to anticancer agents. So, further investigation of the mechanism of p68 promoting cancer development is highly significant for future fundamental and clinical medicine.

## Abbreviations

ER?: Estrogen receptor ?

MyoD: Myogenic differentiation antigen

Runx2: Runt-related transcription factor-2

AR: Androgen receptor

TGF?: Transforming growth factor ?

NPM: Nucleophosmin

ARF: Alternative reading frame

PCa: Prostate cancer

BPH: Benign prostatic hyperplasia

SRC-1/TIF2: Steroid receptor co-activator-1/transcriptional intermediary factor 2

AF-2: Activation function-2

CBP: CREB-binding protein

SRA: Steroid receptor RNA Activator

PSA: prostate specific antigen

c-Abl: Abelson tyrosine kinase

pRb: Retinoblastoma protein

E2F: E2 promoterbinding factor

PDGF: Platelet derived growth factor

Mcl-1: Myeloid cell leukemia-1

MET: Mesenchymal to epithelial transition

EPSC: EMT promoting Smad complexes

PKC: Protein kinase C

SUMO: Small ubiquitin-related modifier

HDAC: Histone deacetylase

COUP-TFI: COUP Transcription Factor 1

HDACI: Histone deacetylase inhibitor

TRAIL: Tumor necrosis factor-related apoptosis-inducing ligand, an anticancer agent

TCF: T-cell factor

HNSCC: Head and neck squamous cell carcinoma

T-ALL: T cell acute lymphoblastic leukemia

SCC: Squamous cell carcinoma

NBNC HCC: Non-hepatitis B virus/non-hepatitis C virus-related hepatocellular carcinoma

## Competing interests

The authors have declared that no competing interest exists.

## Authors¿ contributions

TYD carried out collection and assembly of data, and manuscript writing. LC carried out the collection of data, and helped to draft the manuscript. YSL participated in the collection and assembly of data. ZCY participated in the collection and assembly of data. LT helped to modify the table and figure. XZR conceived of the review and helped to draft the manuscript. CMS conceived of the review and helped to draft the manuscript, and gave final approval of manuscript. All authors read and approved the final manuscript.
